# Towards an Engagement-Aware Attentive Artificial Listener for Multi-Party Interactions

**DOI:** 10.3389/frobt.2021.555913

**Published:** 2021-07-01

**Authors:** Catharine Oertel, Patrik Jonell, Dimosthenis Kontogiorgos, Kenneth Funes Mora, Jean-Marc Odobez, Joakim Gustafson

**Affiliations:** ^1^ Department of Intelligent Systems, Interactive Intelligence, Delft University of Technology, Delft, Netherlands; ^2^ Department of Intelligent Systems, Division of speech music and hearing, KTH Royal Institute of Technology, Stockholm, Sweden; ^3^ Eyeware Tech SA, Martigny, Switzerland; ^4^ Perception and Activity Understanding, Idiap Research Institute, Martigny, Switzerland

**Keywords:** multi-party interactions, non-verbal behaviors, eye-gaze patterns, head gestures, human-robot interaction, artificial listener, social signal processing

## Abstract

Listening to one another is essential to human-human interaction. In fact, we humans spend a substantial part of our day listening to other people, in private as well as in work settings. Attentive listening serves the function to gather information for oneself, but at the same time, it also signals to the speaker that he/she is being heard. To deduce whether our interlocutor is listening to us, we are relying on reading his/her nonverbal cues, very much like how we also use non-verbal cues to signal our attention. Such signaling becomes more complex when we move from dyadic to multi-party interactions. Understanding how humans use nonverbal cues in a multi-party listening context not only increases our understanding of human-human communication but also aids the development of successful human-robot interactions. This paper aims to bring together previous analyses of listener behavior analyses in human-human multi-party interaction and provide novel insights into gaze patterns between the listeners in particular. We are investigating whether the gaze patterns and feedback behavior, as observed in the human-human dialogue, are also beneficial for the perception of a robot in multi-party human-robot interaction. To answer this question, we are implementing an attentive listening system that generates multi-modal listening behavior based on our human-human analysis. We are comparing our system to a baseline system that does not differentiate between different listener types in its behavior generation. We are evaluating it in terms of the participant’s perception of the robot, his behavior as well as the perception of third-party observers.

## 1 Introduction

While the idea of robots being an integral part of our society has been around for many years, it recently became much more immediate. More and more companies are trying to bring companion-like robots onto the market: examples are Pepper, Nao, Jibo, Furhat and many more. The vision is to create socially aware robots that can interact with humans in a human-like manner over long periods of time. For this to be possible, in-depth modeling of human communication to inform human-robot interaction is becoming increasingly important. One important part of human interaction is listening. Listening is arguably more subtle than speaking, but it fulfills equally important functions in human communication. As humans, we not only want to express our thoughts and feelings, but we would also like to feel that we are being listened to and that the listener is taking an active interest in the conversation. To convey that we are listening, humans have developed a set of mechanisms. This set includes looking at the speaker, head nodding, verbal feedback such as “mh,” “okay” and also facial expressions such as frowning and smiling. Appropriately applying them is essential for social interaction to function smoothly. For example, looking at a speaker for too long a time can make a listener appear creepy. Choosing the wrong feedback token, or timing it wrongly can make a listener appear distracted or not listening. Yet, modeling these behaviors is highly complex, not least due to their subtlety and multi-dimensionality. For example, while feedback token such as “mhm” or “okay” can vary in the degree to which they are consciously produced (Allwood, 1993; Kopp et al., 2008; Bevacqua et al., 2010), and despite their limited duration, they have the power to convey a multitude of functions depending on how they are being produced. We humans are very sensitive to noticing whether these feedback tokens are given in a situation-appropriate manner. This might also be the reason why this very subtle human behavior has proven to be important in human-robot interactions (Cassell et al., 1999a; Park et al., 2017; Sidner et al., 2006; Gratch et al., 2006; Skantze et al., 2013). In fact, it has been found that using feedback tokens such as “mhm” or “yeah” as well as head nods inappropriately may have negative consequences for engaging in dialogue (Park et al., 2017). For example, in an interviewing situation, this may negatively affect the perceived performance (Bailly et al., 2016). On the other hand, appropriate modeling positively affects perceptions of empathy and understanding (Kawahara et al., 2016) and rapport (Gratch et al., 2006).

Humans are interacting to a large degree in groups larger than two, and their exchange of social cues includes many modalities. For modeling listening behavior in human-robot interaction, it, therefore, makes sense to use multi-modal cues and to not only focus on dyadic interaction but to include data of multi-party interaction. In addition to feedback behavior also gaze is an important cue to estimate the listener’s engagement in the conversation. Yet, related research on gaze so far has been mainly concerned with the analysis and modeling of dyadic interaction rather than multi-party interaction and has not focused on the role of the listener in particular (Argyle and Cook, 1976; Vertegaal et al., 2001; Vinayagamoorthy et al., 2004; Jaffe et al., 1973; Ruhland et al., 2015; Andrist et al., 2017; Mutlu et al., 2006). Similarily, also research on feedback token has mainly focused on analyzing dyadic interactions e.g., (Kawahara et al., 2016; Ward et al., 2007) and on modeling the timing of feedback token therein (Morency et al., 2010). Which modality should be chosen, audio or visual, and how feedback tokens are combined with gaze has not been the focus of research so far.

To close this gap, this article focuses on investigating the effects of implementing multi-party listener behavior in human-robot interaction. Specifically, we center our research around the question: Does multi-party listener behavior generation in a robot, based on human analysis, lead to an improved perception in comparison to a baseline system? To answer this question, this article to bring together previous analyses of listener behavior analyses in human-human multi-party interaction and provides novel insights into gaze patterns between the listeners in particular. We are implementing an attentive listening system that generates multi-modal listening behavior based on our human-human analysis. We are comparing our system to a baseline system that does not differentiate between different listener types in its behavior generation. We are evaluating it in terms of the participant’s perception, the participant’s behavior as well as third-party observers impressions of the systems.

### 1.1 Paper Structure

This paper is structured as follows. section 2 reviews the literature related to the research question outlined above, specifies explicit hypotheses and lists its contributions. section 3 introduces the multi-party attentive-listener scenario and motivates the evaluation criteria chosen. section 4 describes the human-human data-collection and analysis and its implications for modeling listener behavior in multi-party human-robot interaction. Our underlying assumption is that taking into account human multi-party listener behavior in the implementation of a corresponding model for human-robot interaction will positively affect its perception. section 5 focuses on the multi-party human-robot experiments. Here we describe the task, sensor set-up as well as attentive-listener model implementation in comparison to a baseline model. We discuss our findings in section 6 and conclude this article in section 7, together with recommendations for future work.

## 2 Background

The following sections will provide an overview of multi-party listener modeling in human-robot interaction. It will summarize relevant findings around audio-visual feedback token and eye-gaze in human-human interaction. It will also introduce the construct of social presence and conclude with the formulation of our hypotheses.

### 2.1 Multi-Party Listener Categories

To quantify multi-party listener behavior, we adopt the categorisations of dialogue participant categories that are already established in the literature. As a general framework, we are using the work of Clark. (1996) and Goffman. (1967) for the implementation of such listener categories. Clark. (1996), building on Goffman. (1967), operationalized participation in group interactions as follows. First, a distinction is made between participants and non-participants. The term participant is referring to anyone contributing to and being part of a conversation. This includes the speaker, as well as the current addressee, but can also include further people such as people are part of the group of possible speakers but who currently are taking on a listening role. These participants are being classified as side-participants. In contrast to this is the category of overhearer. Both bystanders and overhearers are part of the non-participant group. A bystander can be defined as a person who the others are aware off, but who does not partake in the conversation. In contrast, an eavesdropper is a person who overhears the conversation without the other participants being aware of it. As these definitions provide a common frame of reference and their usefulness has been shown for related tasks, we decided to build up on these definitions and adapt them for the specific task of listener classification. For this purpose, we have substituted the term addressee for attentive listener, and use the terms side-participants and bystanders to make a distinction to other listener types. For a complete description of the annotation instructions please refer to section 4.2.2. In human-robot interaction some prior work exists around the modeling of participation categories for multi-party interaction. Most related to our work maybe, Matsuyama et al. (2015), who focused on robot interventions to balance speaking time between participants and Short et al. (2016) who investigated the effect of a robot moderator on group cohesion and each participant’s amount of time spent speaking during the conversation. These works have however not been focused on the generation of listener behavior in particular.

### 2.2 Audio-Visual Feedback

In the following sections, we are going to refer many times to back-channels. Yvgne defined back-channels as the “channel, over which the person who has the turn receives short messages such as “yes” and “uh-huh” without relinquishing the turn” Yngve. (1970). Back-channels can convey many communicative functions such as: acceptance, rejection, confirmation or agreement (Allwood et al., 1992). They can, however, also be more ambiguous and simply encourage the speaker to carry on. Such back-channels are often referred to as continuers (Goodwin, 1986). Back-channels are not only restricted to vocalisations but can, and often do, occur in visual or gestural form. Examples of such back-channels are head nods and frowns. Allwood and Cerrato (2003) for example, found that gestural back-channels most often co-occur with vocal back-channels and rarely on their own. Additionally, he points out that back-channels can carry different functions depending on their realization. Listener behavior, including vocal back-channels and head nods, has been explored in dyadic human-agent interaction (Gustafson et al., 2005; Maatman et al., 2005; Gratch et al., 2006; Sidner et al., 2006; Douglas-Cowie et al., 2008; Huang et al., 2011; Schroder et al., 2015) and to our knowledge, at least in one case, also for multi-party human-agent interaction (Wang et al., 2013). Douglas-Cowie et al. (2008) used an artificial listening agent in a wizard-of-oz setting. They provided participants with the option of interacting with different listener personalities (e.g. happy, gloomy etc.). They found this to be a successful method to collect emotionally colored multi-modal interaction data. Schroder et al. (2015) could show that their artificial emotionally expressive listening system had a positive effect on user engagement in comparison to a non-expressive baseline system. They highlighted as one of their main contributions the uniqueness of their system in that it creates a loop between multi-modal human-human analysis, interpretation and affective generation of non-verbal behavior in a human-agent conversational setting. Additionally, they made the software publically available. Maatman et al. (2005) addressed how to use para-linguistic instead of semantic features for improving the responsiveness of artificial listener behavior. Informal tests showed an improvement in the naturalness of the agent’s listening behavior. Huang et al. (2011) showed that accurate modeling of back-channel timing improves the perception of rapport. Yet, none of these studies explored the effects of mono-modal vs. multi-modal back-channels.

Most research on this topic has actually been carried out on human-human interaction focusing on predicting the timing of back-channels (Morency et al., 2010; Truong et al., 2010; Huang et al., 2010; de Kok et al., 2013; Bavelas and Gerwing 2011). Much less work has been carried out on the “what” and “how” to respond. There is some recent work that predicts the morphological form of vocal back-channels (Yamaguchi et al., 2016) and some research investigated how variations in the prosodic realization of a back-channel can change its (multi-dimensional) meaning (Allwood 1993; Poggi 2007; Neiberg et al., 2013). Head nods, on the other hand, have been less investigated (Wagner et al., 2014). Yet, it can be concluded that, like vocal feedback tokens, head nods can have different functions. Those functioning as back-channels are often realized as smaller, single nods (Rosenfeld and Hancks, 1980). Even fewer studies have explored the relationship between visual and verbal back-channels. In a preliminary study, Bertrand et al. (2007) found that when the speaker is looking at his conversation partner, the conversation partner generates a sequence of gestural back-channels. However, the production of gestural back-channels followed by vocal back-channels appeared not to be a common phenomenon during intervals of the speaker looking at his conversation partner. Moreover, Truong et al. (2011) found that head nods are more often produced during mutual gaze. They also found that vocal back-channels are more often produced during pauses in the speaker’s speech.

In a previous study, Oertel et al. (2015) on the same data-set as used in this article we found that head nods occur more frequently than vocal back-channels. We also found that the frequency of both head nods as well as vocal back-channels significantly decreases across listener categories. The ALi category produces the highest number of back-channels and head nods, followed by the SPa and finally the Bys category. Additionally, we found that the likelihood for a vocal back-channel to be produced during mutual gaze is 4.6 times higher than periods of non-mutual gaze. For a head nod, the increased likelihood lies at 2.14. This is a similar, yet less pronounced trend, to that described inBavelas and Gerwing. (2011). The focus of this paper though lies on how the consideration of these results in the implementation of a multi-party attentive listener system, effect its evaluation in comparison to a baseline system.

### 2.3 Gaze Behavior

The fact that it is important to model gaze-behaviour in human-robot interaction has been illustrated by the positive effect it has on the evaluation of the interaction, e.g. Fischer et al. (2015). Research in this domain has mainly been focused on dyadic interaction and there specifically joint attention and turn-taking modeling (Andrist et al., 2017; Yonezawa et al., 2007; Moon et al., 2014; Mutlu et al., 2009b; Skantze et al., 2014; Andrist et al., 2014; Cassell et al., 1999b; Ruhland et al., 2015). While listener modeling for multi-party interaction has not been the focus of research yet its complexity is illustrated by the fact that simply increasing the amount of time gazing at the speaker can make gazing behavior appear strange, scary, or awkward (Wang and Gratch, 2010). This is in line with findings of human-human interaction where it was found that gaze patterns of interlocutors differ when in the role of the speaker or the role of the listener. Typically, the listener gazes at the speaker for longer intervals than vice versa (Argyle and Cook 1976; Vertegaal et al., 2001). In fact, socially acceptable duration of how long to gaze at the interlocutor uninterruptedly can depend on many factors. One such factor is the conversational engagement (Oertel and SalviBednarik et al., 2012; Oertel and Salvi, 2013). Less engaged participants spent more time looking downwards than more engaged participants. There is less work on gaze modeling in multi-party human-robot interaction. Maybe most related to the current work is the work by Mutlu et al. (2009a) who manipulated eye-gaze in a robot to convey to participants which participation role they should be taking on. Additionally, Admoni et al. (2013) found that the duration of gaze fixation of the robot influenced the perceived attention of participants. In a preliminary study Oertel et al. (2015), we investigated the gaze patterns across different listener categories. We found no significant difference in the amount of gaze directed towards the speaker across the different listener categories. However, there was a significant difference in the speaker’s amount of gaze directed towards the different listener categories. There was also a significant difference in the mutual gaze shared between the speaker and the respective listener categories. Additionally, we found that the bystander was gazing downwards more than the attentive-listener. However, this preliminary study was carried out on a limited sample; for this article, we are substantially increasing the sample size and re-analyse the speaker’s gaze patterns and analyze gaze patterns between the different listener categories.

### 2.4 Social Presence and Attentiveness

With our implementation of an attentive-listening system, we are aiming to contribute knowledge towards achieving more socially aware human-robot interactions. When evaluating such a system, it, therefore, makes sense to use measures that capture the perceived social performance of a system. One such measure is the construct of social presence. It is defined as the feeling of “being together with another” (Biocca et al., 2001). It has been correlated with several positive outcome variables such as greater enjoyment (Richardson and Swan, 2003), performance, satisfaction (Biocca et al., 2001; Tu and McIsaac, 2002) as well as trust (Spencer, 2002). Most current work does not address attentive listener modeling for multi-party interaction. By taking the different listener categories into account, we aim at creating this feeling between humans and robots.

While the social presence questionnaire captures the perception of the participant, it does not capture his behavior. Humans are not always aware of their subconscious preferences. Investigating which system the participants pay most attention could contribute further insights.

Social presence is a construct generally used to capture the perception of the participant. As a participant will focus his attention on his task, there is a danger that he will miss out on certain aspects of the behavior generation. Therefore, capturing the impression of third-party observers will provide further insights and maybe better represent the impression of non-speaking participants within a multi-party interaction. A measure that is often used in human-robot interaction to measure the perception of third-party observers and measure participant’s perceptions of a system is the construct of “engagement.” While engagement is defined differently across different studies, one definition that is often used in human-robot or human agent interaction is the one by Sidner defining “engagement as the process by which two (or more) participants establish, maintain and end their perceived connection” (Sidner et al., 2005). Other studies attribute more of an affective dimension to engagement and use engagement more synonymously with “interest” and “attentiveness” (cf. Oertel et al. (2020)). Within the scope of this article, we are using engagement with the latter interpretation in mind. To emphasize this interpretation, we are using the term “attentiveness” within the context of our human-agent and human-robot experiment. We know from the literature that eye-gaze (Oertel and Salvi, 2013; ?), as well as prosody (Oertel et al., 2011), used with an affective interpretation, are important cues for the detection of engagement in group discussions. While, in these studies, no differentiation is made between intervals where a participant is speaking and intervals where a participant is listening, they remain good candidates for the evaluation of an attentive-listener agent for third-party perception.

### 2.5 Hypotheses

We are posing the following hypotheses:

H1: In human-human multi-party interaction, participants will show different gaze patterns depending on their listener category:

a) there will be no significant difference in the amount of time different listener categories gaze towards the speaker
b) there will be differences across listener categories in the amount of gaze received from the speaker
c) the amount of gaze directed downwards will be different between the listener categories
d) between listener categories the bystander, side participant and attentive listener will gaze the most amount of time towards the attentive listener
e) listeners gaze at all participants in an interaction not only the speaker or attentive listener
f) there will be differences across listener categories in the amount of mutual gaze with the speaker

H2: A social robot, that accounts for the multi-party nature of an interaction, by adapting its gaze and audio-visual feedback behavior according to the participatory role of the participant (speaker, different listener categories).

a) will be perceived as more socially present by the speaker
b) will receive more visual attention from the speaker
c) will be received as more attentive by third-party observers than a baseline system that does not adapt its behavior accordingly.

While we acknowledge that feedback tokens and multi-modal back-channels can take on several functions, we restrict our study to the investigation of back-channel tokens in terms of their function in signifying attentiveness. We focus on the indicative types of back-channel tokens that are produced as an unconscious reaction rather than a deliberate response to the speaker, those that have a “continuer” function. A continuer signals to the speaker that he should continue speaking.

## 3 Contributions

Different from previous studies which only focused on listener modeling in dyadic interactions, this article focuses on listener modeling in multi-party settings. We are aiming to close the loop between human-human interaction analysis and human-robot interaction. To achieve this goal, we are carrying out an in-depth analysis of gaze behavior in multi-party human-human interaction. Additionally, we are building upon previous research on the relationship between listener categories and audio-visual back-channels. We are using the gaze and back-channel distributions gathered from the human-human analysis to implement and evaluate an attentive-listener system for human-robot interaction. Our discussion and conclusion section focuses on highlighting the challenges encountered and insights gained in the modeling of both human-human and human-robot data. With this paper, we are aiming to make the first step towards more socially aware listener modeling in multi-party human-robot interaction.

The description of the human-human data set in section 4 and preliminary findings related to H1a,b,c were previously published in Oertel et al. (2015) While initial statistical analysis results seemed promising, the classification accuracy of listener category prediction only rendered moderate success (cf. section 4.5). We suspected a reason for this to be the limited amount of data samples available. For this paper, we, therefore, approximately doubled the data samples analyzed. An exact overview of the additional samples added can be found in Table 1. The analysis presented in this paper under H1 a,b,c, has not been previously published and is based on the added data samples. Additionally, this previous publication was limited to the analysis of gaze patterns from- and towards the speaker as well as gaze directed downwards. It did not include any analysis of gaze patterns between the different listener categories. This analysis is new has been added to the current article (cf. H1 c and d).

**TABLE 1:** The first row of this table states the number of samples per listener categories (LCategory), in absolute numbers and percentages, as originally reported in Oertel et al. (2015). The second row of this table states the numbers as used in this article. It can be observed that the number of samples got more than doubled and that in 82.0% of cases, a majority annotation was reached. Ties occurred mainly between neighboring categories such as ALi-SPa and SPa-Bys, as opposed to further away categories such as ALi-Bys.

	Ali	SPa	Bys	ALi-SPa	SPa-bys	ALi-bys	No maj
Amount	62 (16.1%)	82 (21.2%)	118 (30.6%)	21 (5.4%)	37 (9.6%)	7 (1.8%)	59 (15.3%)
Amount	138 (16.5%)	216 (25.9%)	240 (28.7%)	31 (3.7%)	48 (5.7%)	12 (1.4%)	150 (18.0%

In section 5.1 we are summarizing the results of a pre-test that served to exclude confounding factors that might have influenced our human-human analysis. It also served as a bridge between the human-human and human-robot experiments and provided further insights into the perception of multi-modal back-channel realisations. This pre-test was previously published in Oertel et al. (2016). As we are using its findings for our attentive-listener system, we are summarizing it in this article for the ease of the reader.

With the experiments designed to answer H2, we are contributing a whole new set of experiments designed to understand the effect human-behaviour inspired feedback generation has on the perception of multi-party human-robot interaction.

## 4 Learning From Data: Human-Human Data Collection

This section describes the data-set we used for our human-human analysis. It provides details on our procedure of listener category annotations and explains the feature extraction methods used for our subsequent analysis. We report results on gaze patterns within listener categories and between listener categories. We conclude this section by commenting on the usefulness of the extracted features for the automatic prediction of listener categories and summarize the most important findings regarding the subsequent human-robot experiment.

### 4.1 Data Description

**FIGURE 1:**
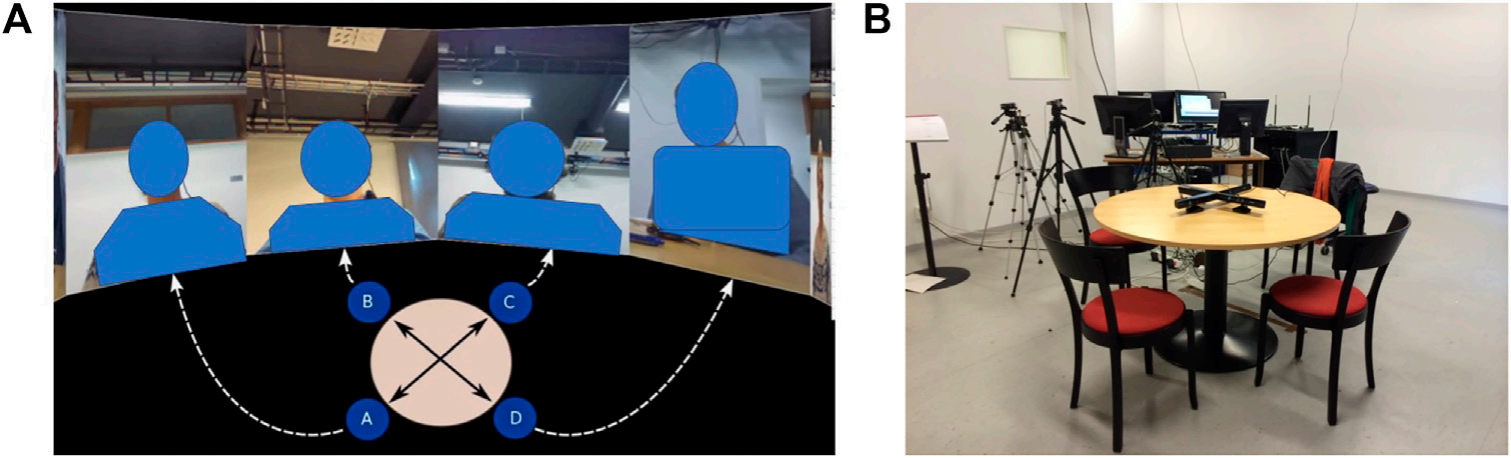
Depictions of the KTH-Idiap Corpus. **(A)** Video Clip Arrangement. Annotation material. Annotators were given a 15 s video slice, as illustrated above. **(B)** Corpus Collection Set-up.

To collect suitable data, we used a corpus of four-party interactions (Oertel et al. (2014)). The interactions consisted of interviews. One participant, the moderator, interviewed three other participants, who competed on winning a scholarship. Participants were first asked to introduce themselves, then describe their PhD work along with how they saw their work could impact society at large, and finally design a joint project. After the initial introduction, the moderator let participants talk and remained in the role of a listener for long stretches of the interaction. Similarly, given the nature of the interaction participants also took on the role of a listener for larger durations of the interaction. Five sessions were recorded. Each session lasted for about an hour. The set-up is shown in Figure 1B. Participants behaviors were recorded using a range of individually synchronized sensors. These include head-mounted microphones, Microsoft Kinect one sensors that are positioned at a distance of around 0.8 m away from participants, and high-resolution GoPro cameras.

### 4.2 Annotations

To conduct our analysis, we needed annotations about the listeners in our dataset. We, therefore, adopted a thin slice approach (Ambady et al., 2000) for which we extracted video clips, including at least one listener from the corpus. These clips were given to the annotators for annotations. The duration of the video was a compromise between providing sufficient information to annotators to form an opinion but not a too broad range of behaviors to cause confusion. In line with prior work on engagement by Bednarik et al. (2012) and interest-level inference by Gatica-Perez et al. (2005), we decided on a video-segment-length of 15 s.

#### 4.2.1 Video Segments

An illustration of how videos were displayed to annotators is given in Figure 1A. Annotators were provided with a view of all four participants. We aimed at reproducing the feeling of a 3D setup as much as possible in order to facilitate the observation of participant’s visual focus of attention. An additional top-view table illustration was added to the bottom of the video to further aid annotator’s grasp of geometric relations. It has to be noted that all videos were presented without audio-track. The reasoning behind leaving out the audio-track was to exclude the influence of semantic speech content on annotations and to allow annotators to concentrate fully on the visual content. Additionally, the lack of audio helped to disguise the moderator’s role. Besides, focusing on low level rather than higher-level features allows for more robustness in real-time systems. However, this choice admittedly brings with it the limitation that modeling remains on a surface level.

#### 4.2.2 Coding Instructions

We asked annotators to provide a listener type categorization for each video clip. Annotators were given definitions of three listener types as listed in the paragraph below and already described in Oertel et al. (2015):

• Attentive Listener (ALi): An attentive listener is the person who is most likely to start speaking after the current speaker.
• Side Participant (SPa): A side participant is a person who is part of the group of potential future speakers, but is probably not the next speaker.
• Bystander (Bys): A bystander is a person whom the group of potential future speakers is aware of, but whom they do not expect to speak in the near future. The bystander acts as an observer rather than a potential future speaker.”

Participants were asked to watch the video clip at least twice before deciding on an annotation. Specifically, we asked them to focus on the indicated participant when watching the clip for the first time, and the group, when watching the clip for the second time. Our complete pool of annotators comprised 25 people, mainly sampled from a post-graduate population. All participants were naive to the task. We provided all of them with five video clips for training, that were afterwards excluded from the overall analysis. These training video clips showcased a range of behaviors to help the annotators get used to the kind of behaviors they could expect. Each annotator spent approximately 40 min on the annotation task. All annotations were carried out voluntarily without any compensation granted. Preliminary findings have previously been reported by Oertel et al. (2015).

#### 4.2.3 Relationship to Engagement

In Oertel et al. (2015), we also investigated the relationship between the listener category annotation with the construct of engagement. Engagement (ENG) was rated by third party observers on a seven-point Likert scale from 1 (not engaged) to 7 (highly engaged). ANOVA tests revealed that the listener categories affected ENG F (4,314) = 114.8 (*p* < 0.001)).

#### 4.2.4 Video Sampling

It was not possible to have all 5 h of data being annotated. To still cover as much variability in behaviors as possible, we automatically assigned each participant to be either of two roles: the speaker (Sp), or listener. Based on the duration that had passed since the last time they spoke and the start of the video clip window (denoted LT), we further distinguished between two listener types; listener type A (La) and listener type B (Lb). The moderator was mostly incorporated in the (Lb) category.

If it was more than 20 s, (Lb)was assigned and if it was less Lawas assigned. We then assigned video clip categories according the role combinations as stated below: (Sp,La,La), (Sp,La,Lb), (Sp,Lb,Lb), (Sp,Sp,La), (Sp,Sp,Lb). In accordance with these definitions, and the following rules, 600 video-clip windows were sampled. In total we defined three rules. 1) no window was included in which speakers spoke for less than 3 s; 2) video-clips windows were sampled uniformly across all category types and interviews; 3) the decision on which of the participants to annotate was made uniformly among the pool of participants, including the moderator. Most video-clips were annotated by four annotators, however, some video-clips also received three or five annotations. The minimum number of annotations we accepted per video-clip was 3. For each window video-clip at least three annotators had to agree on a listener category (ALi, SPa or Bys) for it to receive that annotation. We assigned an in-between category if there was a tie between two categories (2–2). If each of the annotators chose a different category, the “no majority” category was assigned. Results are presented in Table 1. It can be summarized that using visual information alone suffices to reach a majority vote in the vast majority of cases.

### 4.3 Data Processing

To study the dynamics which relate to the different listener categories, we processed the corpus to extract relevant audio and visual cues. These include speaking turns and vocal back-channel, head nods and gaze patterns.

#### 4.3.1 Back-Channels

Back-channels were automatically annotated following the definition of very short utterances being surrounded by the speech of another participant (as proposed by Heldner et al. (2011)).

#### 4.3.2 Gaze Coding

Gaze was annotated automatically using the method described in Oertel et al. (2015). The approach takes the RGB-D data from the Kinect as input to: 1) constructs a 3 days model of the person’s face; 2) uses the iterative closest points (ICP) algorithm to register the face model onto each depth frame and thus estimating the 3D head pose; 3) extracts rectified eye images which are then passed through a machine learning model to retrieve the gaze direction, and finally; 4) based on the 3D gaze of each person and their relative positions, it was computed at whom each participant was looking at. Moreover, if a person was found not to be looking directly to another participant, the approach classified the gaze as otherwise looking “up,” “down” or “others” by simply monitoring the 3D gaze ray in relation to all other participants.

#### 4.3.3 Head Nods

The annotation of visual back-channels, i.e. head nods, was conducted automatically based on the work of Nguyen et al. (2012). Given that the 3D head pose was available from the tracking algorithm described in the previous subsection, Gabor filters were computed from the pan, tilt and roll signals. Then, an SVM classifier, using a radial-basis function, was used to detect the head-nods from the Gabor filter features. As found by Nguyen et al. (2012), the method was effective to infer nods mostly from listeners whereas it was found to be less accurate for speakers.

#### 4.3.4 Higher-Level Cues for Listener Characterization

In order to analyze how the different low-level features are related to the listener categories we followed the same methodology as described in Oertel et al. (2015). Means and standard deviations of low-level cues were calculated for each participant and each clip and significance tests were carried out cf. section 4.4. Definitions of calculated cues are taken from Oertel et al. (2015) and included in the next paragraph for the convenience of the reader.

• “Visual back-channels” (VisBack): the number of nods detected during the window duration.
• Vocal back-channels (AudioBack): the number of verbal back-channels uttered by the listener during the window duration.
• Gaze at speaker(s) (GazeAtSpeak): percentage of window frames in which the listener looks at the person currently speaking.
• Gaze received from speaker(s) (GazeFromSpeak): percentage of window frames in which the actual speaker looks at the listener.
• Mutual gaze with speaker (MutGazeSpeaker): percentage of window frames in which the listener and the current speaker look at each other.
• Gaze down (GazeDown):percentage of window frames in which the listener looks down/in front of himself/herself.

### 4.4 Results

Using the set of features described in the previous section, in this section, we are testing hypothesis H1 and H2 (cf. section 2) which states that there are differences in listener relevant behavioral cues in humans. If there are such differences, then we can use this information to inform the algorithms implementing our data-driven artificial listener. While initial results have already been reported elsewhere by Oertel et al. (2015), the number of annotations has been extended (approximately doubled) and differences are reported. The ANOVA analysis which will be reported here is based on the extended annotations. Additionally, we are reporting a whole new set of analyses that focus on differences in gaze-patterns between the different listener categories.

#### 4.4.1 Gaze Patterns in Relation to Speaker

**FIGURE 2:**
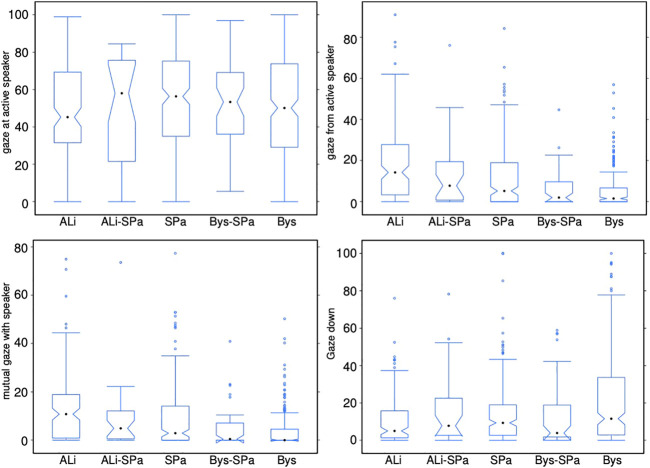
Eye gaze patterns between the various listener categories and the speaker as well as downwards. See section 4.3.4 for the definition of these measures.

Figure 2 illustrates the listener’s gaze patterns in relation to the speaker as well as gaze aversions downwards. A Kruskal-Wallis test provided very strong evidence of a difference (*p < 0*.*001*) between the mean ranks of at least one pair of groups. Dunn’s pairwise tests were carried out for the three pairs of groups (significance tests between pairs of groups are reported in Table 2). There was very strong evidence (*p < 0*.*001*, adjusted using the Bonferroni correction) of a difference between the listener categories SPa and Bys and a strong difference (*p < 0*.*01*) between ALi and SPa in the amount of gaze the received from the speaker. The ALi received a significantly more proportionate amount of gaze from the speaker than the SPa, and the SPa more than the Bys. There was also very strong evidence (*p < 0*.*001*) of a difference between the listener categories SPa and Bys and evidence of a difference (*p < 0*.*05*) between the ALi and SPa in the amount of mutual gaze. The ALi shared significantly more mutual gaze with the speaker than the SPa and the SPa significantly more than the Bys. Additionally, our analysis found strong evidence (*p < 0*.*01*) of a difference between the listener categories ALi and Bys in the amount of gaze directed downwards. The Bys averted his gaze downwards for significantly longer times than the ALi. It can also be observed that the listener gazes more at the speaker than the other way around. This finding supports Argyle and Cook. (1976) previous finding.

**TABLE 2:** The first row of this table states the results of the posthoc test, as originally reported in Oertel et al. (2015). The second row of this table indicates the results of the posthoc test after having added the extra samples specifically for this article. It can be observed that the significant differences between listener categories did not change.

Categories	Gaze_At_Speak		Gaze_From_Speak		MGaze_Speaker		Gaze_Down	
	ALi_SPa	SPa_Bys	ALi_SPa	SPa_Bys	ALi_SPa	SPa_Bys	ALi_SPa	SPa_Bys
Original results	NS	NS	*p* < 0.001	*p* < 0.001	*p* < 0.001	*p* < 0.01	NS	NS
Updated results	NS	NS	*p* < 0.01	*p* < 0.001	*p* < 0.05	*p* < 0.001	NS	NS

#### 4.4.2 Gaze Patterns Between Listener Categories

**FIGURE 3:**
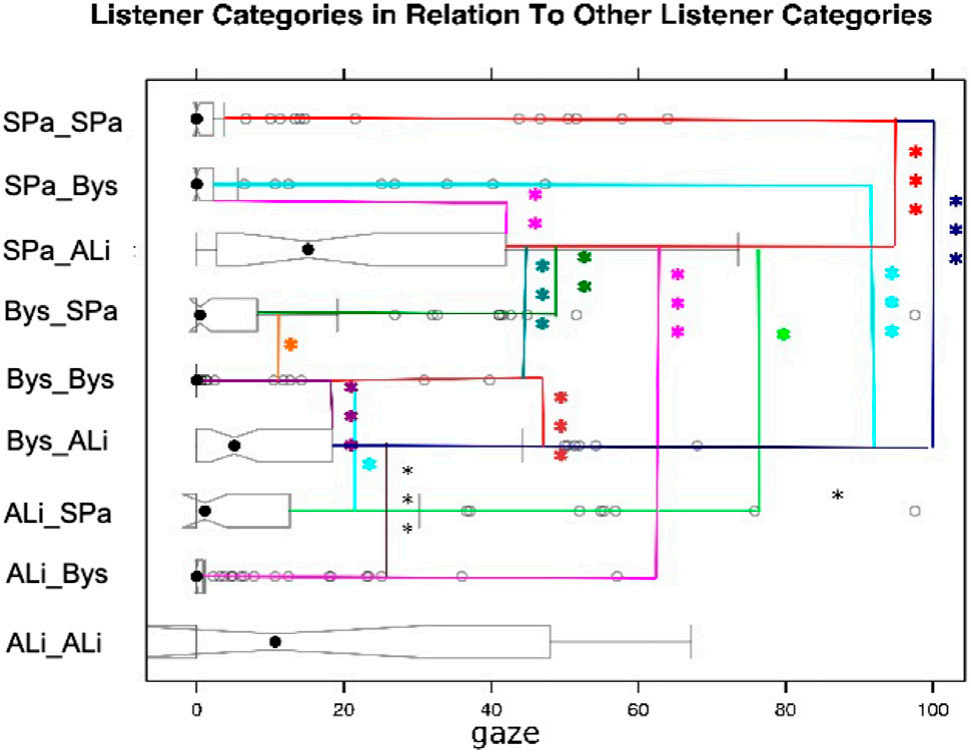
Gaze patterns between listener categories.

When modeling multi-party listener behavior, it is essential to understand gaze distribution between the speaker and the different listener categories. However, given the multi-party nature of the interaction, listeners may not only direct their gaze towards the speaker but also towards different listeners. Therefore, it is important also to investigate these distributions. Figure 3 illustrates the gaze distributions between the different listener categories. Please note that while it was not a frequent case, it did happen that two different participants in a video got assigned the label of ALi by third-party observers. Similarly also the assignments of two SPas or two Bys is possible. A Kruskal-Wallis test provided very strong evidence of a difference (*p < 0*.*001*) between the mean ranks of at least one pair of groups. Dunn’s pairwise tests were carried out for all listener categories. There was very strong evidence (*p < 0*.*001*, adjusted using the Bonferroni correction) of a difference between the listener categories SPa_SPa and SPa_ALi, SPa_SPa and Bys_ALi, SPa_SPa and SPa_ALi, SPa_ALi and Bys_Bys, SPa_ALi and ALi_Bys, Bys_Bys and Bys_ALi, Bys_ALi and ALi_Bys. There was strong evidence *p < 0*.*01* of a difference between the listener categories SPa_Bys and SPa_ALi, SPa_ALi and Bys_SPa. There was evidence *p* < 0.05 of a difference between the listener categories SPa_ALi and ALi_SPa, Bys_SPa and Bys_Bys, Bys_Bys and ALi_SPa. We can observe that listeners direct their gaze to a higher proportion to the more engaged listener in the scene. For example, a side-participant is more likely to gaze at an attentive listener than a bystander. Similarly, a bystander gazes more at an attentive listener than the other way around.

### 4.5 Listener Category Prediction Experiments

We carried out several machine learning (ML) experiments, that are detailed in greater detail in Oertel et al. (2015), to investigate the possibility of automatically predicting listener categories from the data gathered and analyzed above. Given the limited data available, we decided against the use of deep learning but tested both decision tree and support vector machine approaches instead. Balancing the data-set per listener category, the highest average accuracy achieved was 54.1%, using support vector machines. The addition of additional data points, see table 1, did not lead to an improvement in prediction accuracy. While beyond the scope of this paper, we believe that the accuracy could be improved if additional content related cues were added. For evaluating our attentive listening system, we, therefore, decided not to add an ml-based module to our system but to use a rule-based approach that draws from the collected gaze and back-channel distributions per listener category. We describe the process in further detail in section 5.4.1.

### 4.6 Summary of Multiparty Human Listener Modeling

We found that there were no differences in the amount of gaze directed towards the speaker across the different listener categories. However, we did find differences in the amount of time the speaker gazed towards the different listener categories, i.e., H1 a and b can be confirmed. We could not find significant differences in gazing downward between the attentive listener and side participant and side participant and bystander. We only found differences between the attentive listener and bystander. Therefore, we can only partially accept H1 c. H1 d can be accepted as we did find that the bystander, side participant and attentive listener gazed the most amount of time towards the attentive listener. H1 e can also be accepted as the listeners gaze at all participants in an interaction not only the speaker or attentive listener. Finally, we can also accept H1 f. We found that the speaker shared more mutual gaze with with the attentive listener than the side participant and more mutual gaze with the side participant than the bystander.

## 5 Multi-Party Human-Robot Experiments

This section focuses on our proposition of an artificial listener model for multi-party human-robot interaction. We start this section by summarizing the results of a pre-test that evaluated the perception of different audio-visual back-channel realisations in an agent. We then continue to detail the experimental setup. We describe our baseline and the attentive-listener system implementation that is based on our statistical analysis of multi-party human-human interactions. We provide information relating to two studies. The first focuses on the perception and behavior of the study participants, and the second focuses on perception of third-party observers. We conclude this section by reporting on the respective results elaborated on in the subsequent discussion section.

### 5.1 Pre-Test:Perception of Back-Channel Generation in a Virtual Agent

To test the effect on the perception of attentiveness by generating head nods and back-channels in a robot, we decided to carry out a pre-test before starting the complete human-robot experiment. This pre-test was previously published in Oertel et al. (2016), and we are only summarizing its results here for the reader’s ease. Using the pre-test methodology allowed us to exclude other variables that might influence the perception of a back-channel or head nod (such as the appearance of the speaker or his/her facial expressions). For this, we decided to replay the original participant’s back-channels and regenerate their head nods in a virtual agent. Specifically, we extracted sentences in which a back-channel occurred across the whole data collection. These sentences would serve as carrier sentences. To avoid effects caused by incorrect timing, newly generated back-channels were added at the exact point in time where the original back-channel had occurred. This means that the virtual agent back-channels against the original speaker’s speech. We recorded videos of the virtual agent generating the audio-visual back-channels. These videos served as stimuli for our pre-test perception experiment. Specifically, we set up a task on the crowd-working platform crowdflower. Crowd-workers were presented with the same video of a turn-segment twice. The only difference being the realization of the back-channel. Crowdworkers were asked through pairwise comparisons to indicate in which video the virtual agent showed more, respectively less attentiveness. We were both interested in identifying discriminative head-nod related features and comparing uni-modal (just verbal back-channel or audio back-channel) to multi-modal back-channel generation.

Relevant to this article is that we could indeed show that a human can perceive differences in an agent’s perceived degree of attentiveness related to the generation of audio-visual back-channels.

Specifically, we could show that “maximum downward amplitude,” “frame duration,” “the number of oscillations,” “first amplitude” and “maximum downward speed” were discriminative between less and more attentively perceived head nods.

With regards to vocal back-channels we found rms to be a discriminative feature. We found differences in the way attentiveness is expressed in mono-, in contrast to bisyllabic back-channel token. We could also show that head nods were generally perceived to convey more attentiveness compared to audio back-channels. However, the fusion of modalities appeared to increase the degree of perceived attentiveness. We were able to automatically rank head-nods with an average accuracy of 74.3%. For full details on the statistical analysis, please refer to Oertel et al. (2016). These findings enabled us to improve upon the human-robot experiment’s design that we will detail in the next section.

### 5.2 Task

This section describes the task around which we designed the human-robot interaction, the robot used in our study, its perception capabilities, its attentive listener model and detail the role of the wizard.

#### 5.2.1 Task Content

The human-robot interaction scenario revolved around a storytelling interaction. This constituted a change from the human-human data collection scenario and was done to optimize for the evaluation of the robot’s attentive listening capabilities. Participants were given three tasks in total. First, they were asked to retell the story of Cinderella. Afterwards, they would be asked to talk about their favorite movie. Finally, they were asked to fill out the social presence questionnaire and to indicate whether they perceived one of the robots to be more attentive.

#### 5.2.2 Roles

Each interaction included one moderator, two social robots and one participant. The moderator’s role was to start the experiment. He first introduced himself. He then asked the two robots to introduce themselves and also asked the participant to do the same. After the introductory phase concluded, the moderator asked the participants to start with retelling the Cinderella story. He reminded them to ignore him for the duration of this task and to focus on identifying which of the robots was paying attention to them. He also informed them that he would address them again once it was time to continue to the movie retelling part of the experiment. The two social robots followed the moderator’s instruction and introduced themselves, using their respective names, Joakim and Gabriel (To help the participant differentiate between at a later stage a name tag was also attached underneath their respective head). They elaborated that they were developed at the department of speech music and hearing at KTH and that they are each other’s brother. The robots contributed to the storytelling scenario through active listening. It is made clear that the robots do not have any further dialogue capabilities apart from this. The robots demonstrate their active listening through a combination of gaze and audio-visual feedback behavior.

#### 5.2.3Robot Embodiment

We used two back-projected robot heads for our experiment. Their only differentiating features were their hat and the name-tag attached underneath the robot head. They were using the same voice. Our decision for the use of back-projection technology was made because of its capability of realizing fine-grained gaze changes in quick succession. Additionally, the physical servos of the robot’s neck allow for realizing head nods and lip synchronization for audio-backchannels could be insured. Accurate and detailed non-verbal behavior realization is essential for a task such as ours, where a human must be able to perceive even subtle gaze changes and be able to identify the robot’s gaze target undoubtedly.

### 5.3 Sensor Set-Up

The following section comprises information relating to the room set-up as well as multi-modal perception set-up. By multi-modal perception, we are referring to the motion-tracking, audio and video information used to provide our systems with sensory information. We are here also providing detail on the role a wizard takes in our system implementation.

#### 5.3.1 Room Set-Up

**FIGURE 4:**
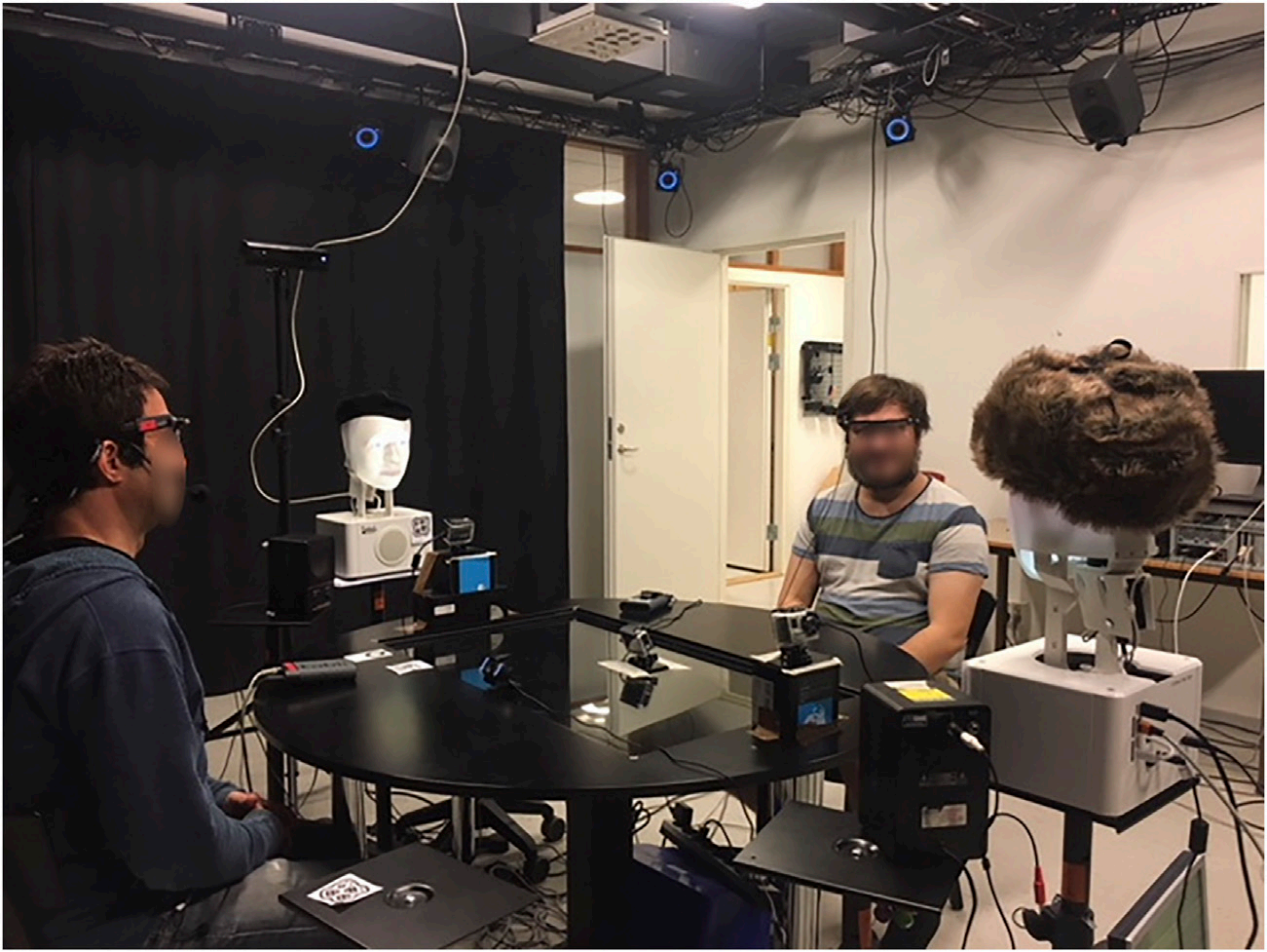
The experimental setup for the evaluation of the proposed data-driven artificial listener, in comparison to the baseline system.

Figure 4 illustrates the room set-up that was optimized to facilitate multi-party interaction. There were two back projected robot heads and two humans sitting around a round table. The two robots are facing each other, and the two humans are facing each other. The moderator sat across the table from the human participant. The participant sat with his/her back towards an adjacent experimenter room with a connecting window. Particular attention was given to create equal distance between participants and robots so that it would require equal effort to look at each other. Additionally, it was ensured that a gaze shift would not suffice to look at a participant to the left or the right, but that the head would need to be shifted to achieve this. The choice of exposing the participant to two robot systems was taken in order to facilitate comparison making for the participant.

#### 5.3.2 Multi-Modal Perception

We placed a go-pro camera in front of both humans and robots for a close-up face shot. Additionally, there were also cameras installed behind both humans and robots to enable a more global room view from the view-point of the respective participant. We used one additional camera to capture the whole room. These video recordings were used for subsequent analysis alone. Both moderator and human participant were equipped with close-talking microphones. This audio-feet was used to estimate speaking in real-time. Participants and moderator were equipped with glasses to which we attached motion-tracking markers. Using our motion tracking set-up, we were able to infer participants head rotation. By defining head rotation intervals for a given gaze target, we could automatically and in real-time infer whether the participant was looking at either of the robot-participants, the moderator or “elsewhere.” Before the start of the experiment, we did pilot tests to ensure that the head-tracking was a good approximation for the visual focus of attention, which, also thanks to the room set-up, it was. Synchronization was ensured through the use of a clapper that had motion tracking markers attached to it. We clapped it in front of the room overview camera. The motion tracker signal was used for synchronization with the system logs, whereas the visual channel of the clap was used for audio and video synchronization.

#### 5.3.3 Wizard

As discussed in the background section, the perception of a conversational system is determined by both the accuracy of timing and appropriate choice in back-channel function and realization. To allow us to investigate the latter, it was therefore essential to ensure the accuracy of timing. We achieved this by employing a wizard who had audio-visual access to the experimental room. She was tasked to indicate feedback-relevance-points (FRP) by pressing a button. FRPs are points within the interaction at which it is appropriate to back-channel. They were provided to both the attentive listener system and the baseline system in real-time. Whether they were taken up or not was decided autonomously by the respective systems.

### 5.4 Robot Behavior Implementation

**FIGURE 5:**
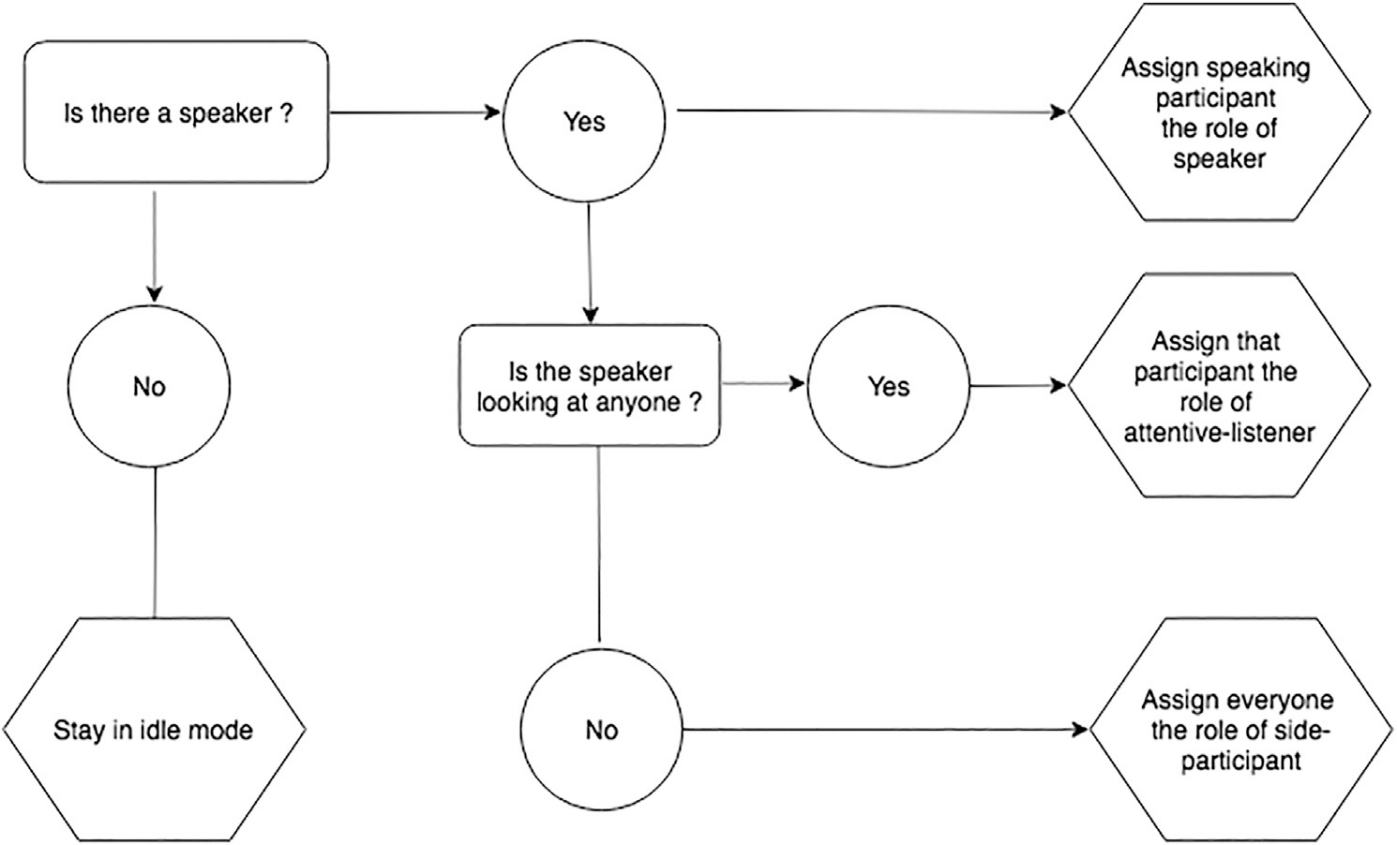
Rules for assigning listener categories: General case.

**FIGURE 6:**
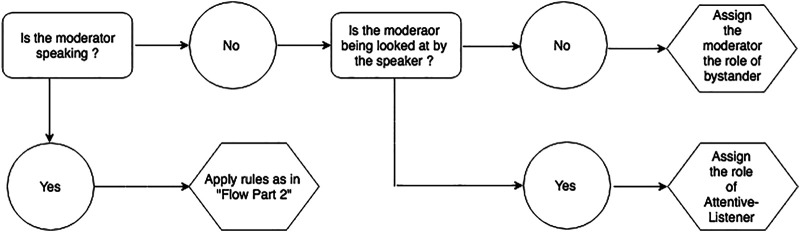
Rules for assigning listener categories: Moderator case.

The robot’s feedback behavior implementation is based on the real-time multi-modal perception data, cf. section 5.3.2 as well as the wizard’s indication of feedback-relevance points. Participant’s influence the robot’s attentive listening response through their gaze behavior and speech activity. All perception data is calculated in an infinite loop over 15-s intervals. It is amended if the listener category changes. A listener category is only changed if a robot is being gazed at for more than 2 s. This threshold has been established through experimentation, also taking into account the robots affordances. The robot’s listening behavior is dependent on its category assignment that is determined by five rules. See Figure 5 and Figure 6 fora flow diagramme depicting the rules and Table 3 for a description of the rules.

**TABLE 3:** Rules for listener category assignment.

Rules	Description
Rule 1	If voice activity is detected, then the participant gets assigned the role of the speaker
Rule 2	The robot being gazed at gets assigned the role of attentive listener
Rule 3	The robot not currently being gazed at is assigned the role of side-participant
Rule 4	The moderator can also be assigned the role of bystander. He gets assigned that role if he is not speaking and not being gazed at. Otherwise, he gets assigned the role of attentive listener or speaker respectively
Rule 5	If no speaker is detected then the robot remains in idle mode


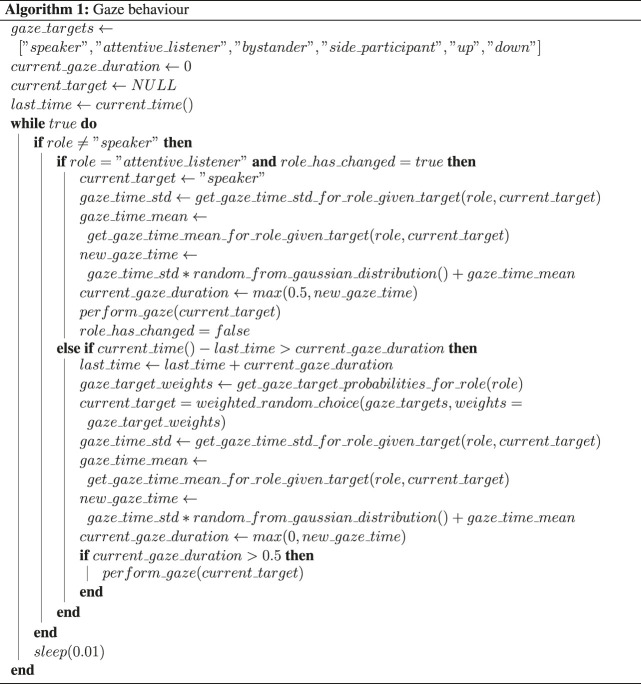


#### 5.4.1 Attentive Listener System

The attentive listener behavior realization is based on the multi-modal data human-human collection and subsequent multi-modal analysis, as described in section 4.4. Gaze and feedback behavior generation is dependent on the listener category assignment, and distributions are extracted per 15-second-intervals.

Gaze behavior implementation includes decisions at which of the three possible gaze targets (moderator, participant, second robot) to look at and for how long. This also includes the modeling of short gaze aversions at the same target. The pseudocode for gaze behavior generation is described in Algorithm 1.

Feedback behavior generation is divided into audio back-channel and head nods. While a wizard provides the feedback-relevance points, the decision whether to produce a back-channel or not at this point is taken by the system. Decisions are also taken on whether to produce a head nod or an audio back-channel. Frequency distributions of head nods and back-channels are taken from our previous study on the same corpus Oertel et al. (2015). The findings of our pre-test 5.1 relating to subtle differences in head nods and back-channel realisations further contributed to the feedback behavior design cf. section 2 ? Specifically, We restricted the use of back-channels to continuers (back-channels that indicate to the speaker to continue talking) only, and we used two levels of continuers. These two variants were associated with more and less attentiveness, respectively. More attentively perceived back-channels tokens had a greater duration of the second syllable, a smaller F0 slope for the first syllable and higher rms intensity of both syllables.

The processes for head nod generation and gaze behavior realization ran as an infinite loop and in parallel to the rest of the system.

#### 5.4.2 Baseline System

As in the attentive-listener system, the baseline system follows the five rules, outlined above for listener category assignment. However, in contrast to the attentive-listener system, the baseline system uses a flat distribution for modeling its gaze and feedback generation. Two exceptions apply. First, when a speaker looks at the robot running the baseline system for more than 2 s, the robot reciprocates by gazing back at the speaker. Second, as in the attentive-listener system, the baseline system was also provided with the feedback relevance points. This means that the random distribution of feedback behavior only applied within the range of possible feedback points.


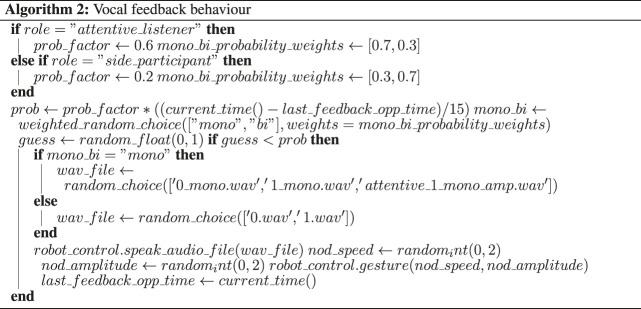



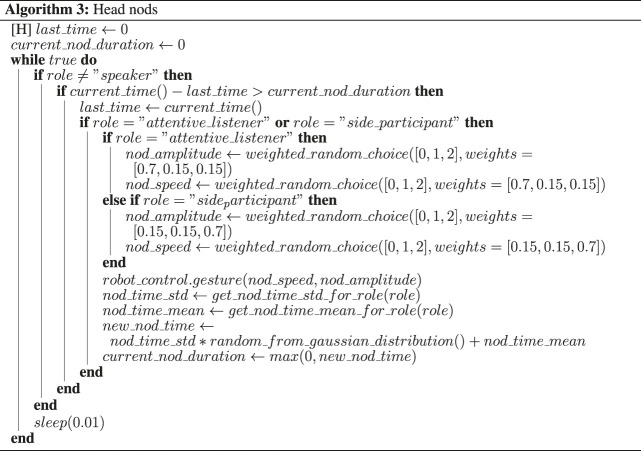


### 5.5 Study I

In this section, we are carrying out two studies that are both related to the participant. In the first study, we are investigating his perception of the robot’s social presence. In the second one, we are investigating at which robot system he spends more time gazing.

#### 5.5.1 Participants

We recruited a total number of 12 participants (9 male, three female)from a pool of students and staff at the Royal Institute of Technology, KTH via e-mail advertisement. Participants received a cinema voucher in exchange for their participation in the study.

#### 5.5.2 Procedure

Before the conduction of the experiment, participants were briefed about the study procedure and asked whether they felt comfortable with the study set-up. Specifically, they were told that they would be greeted by a moderator who would introduce them to two robots and that the experiment would be split into two parts. In the first part, they would be asked to retell the Cinderella story, and in the second part, they would be asked to talk about their favorite movie cf. section 5.2.1. They were tasked to find out whether 1, two or none of the robots was paying attention to them. They were informed that the interaction would be audio and video recorded and that some sensor set-up and synchronization steps would be necessary cf. section 5.3.2. They were informed that we would be asked for their consent to use the recordings for analysis once more after the recording had taken place. They were informed that the experiment would conclude with them filling out a questionnaire. Once they agreed to the procedure, they were brought into the study room, and the experiment commenced. In each interaction, one robot system was using the baseline system, and one robot system was using the attentive-listening one. We were exchanging for each interaction which system controlled the robots’ behavior. This means that in 50% of the total of all interactions, the attentive-listening system was controlling the robot situated to the left of the participant and in 50% the robot situated to the right of the participant.

#### 5.5.3 Method I: Participant’s Impressions of Social Presence of the Robots

To evaluate the model, we first collected participant’s impressions of the robots in terms of their social presence. For this, we chose the Social Presence Questionnaire (Harms and Biocca, 2004). The social presence questionnaire captures the following dimensions: textitCo-Presence, textitAttentional-Allocation, textitPerceived Message Understanding, textitPerceived Affective Understanding, textitPerceived Behavioral Interdependence and Perceived Emotional Interdependence. We used the first five items of the social presence questionnaire. We exchanged the last item, Perceived Emotional Interdependence, for a direct question concerning attentive listening. We decided to do so as we were not manipulating the mood of the robot but the perception of attentiveness. Similarly to Pereira et al. (2019), we divided the social presence questionnaire in two directions: perception of self and perception of the robot. The questionnaire contained a total of 30 items (6 items per category), which the participants answered on a five-point- likert scale. The likert scale ranged from 1 (Strongly Disagree) to 5 (Strongly Agree). To differentiate between the two robots in the questionnaire, they were referred to with their respective name, Joakim and Gabriel.

#### 5.5.4 Method II: Participant’s Focus of Attention

We also evaluated our system in terms of visual attention received from the speaker. The reason for this was that in our human-human data collection, we found that the speaker looked more at the attentive listener speaker than the side-participant or bystander. Following the same approach as described in the human-human analysis, we divided the interaction into thin-slices of 15 s each. For each of these thin-slices, we calculated participant’s focus of attention towards the attentive-listening system and the baseline system. We used participant’s head rotation as a proxy for estimating their focus of attention towards each of the robots. The validity of head rotation as a proxy was facilitated through the room set-up and evaluated via a pilot-test.

#### 5.5.5 Results I:Participants Perceptions of the Social Presence of the Robot

**FIGURE 7:**
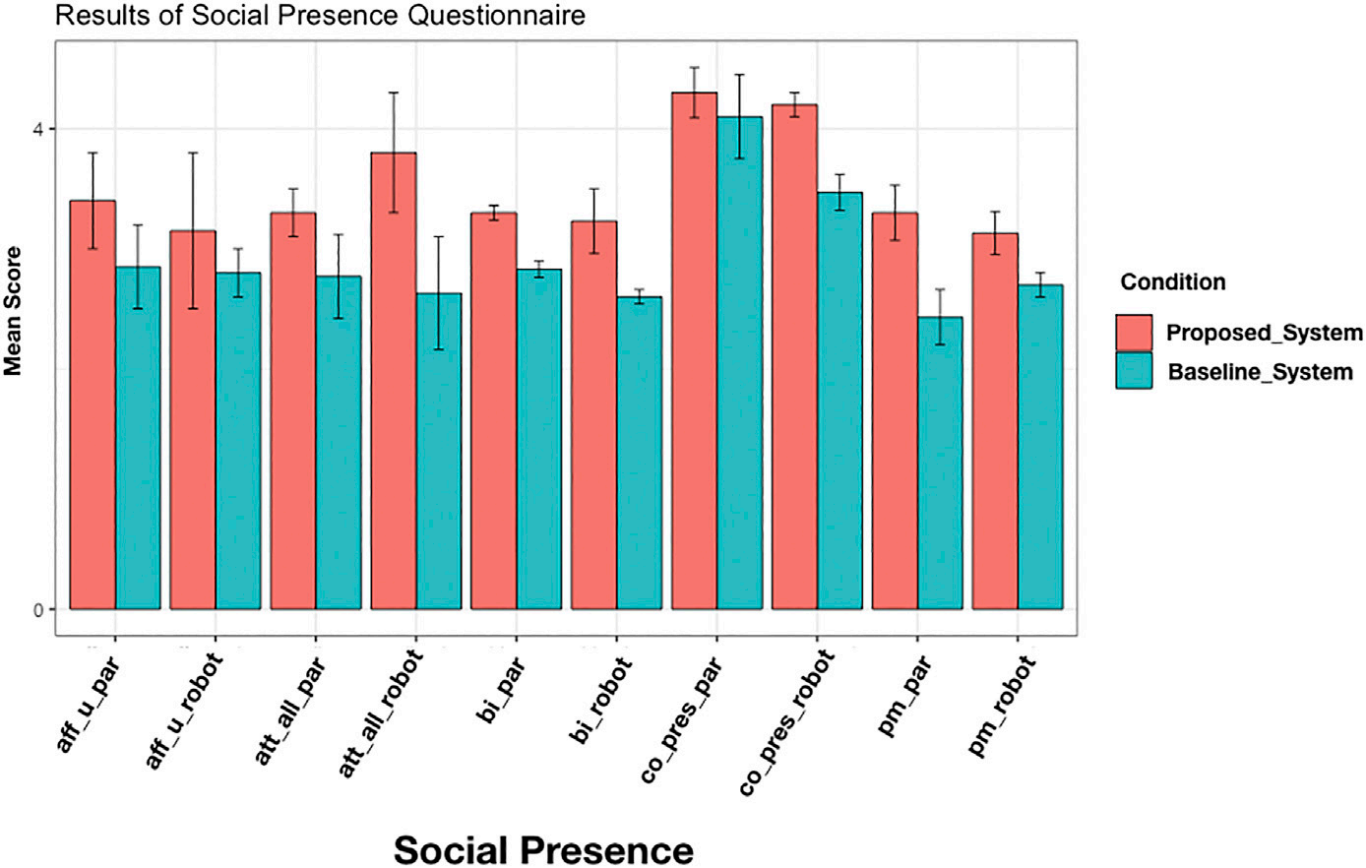
Results of Social Presence Questionnaire. Mean values for each direction of the five different social presence dimensions (Harms and Biocca, 2004)._par indicates the direction of the participant. _robot the direction of the robot (aff_u) Perceived Affective Understanding, the ability to understand the other’s emotional and attitudinal states (Att _all) attentional allocation, the amount of attention the user allocates to and receives from the other (bi) perceived behavioral interdependence, the extent to which the user’s behavior affects and is affected by the other’s behavior; (co_pres) co-presence, the degree to which the observer believes s/he is not alone (pm) perceived message understanding, the ability of the user to understand the message from the other.

The results of the Social Presence Questionnaire are summarized in Figure7. Each dimension of the Social Presence Questionnaire contains questions relating to the perceived perception of the robot and separately human. In the analysis, we separated the two directions. It can be summarized that the attentive listener system was perceived more positively in all five dimensions than the baseline system. As this article’s focus is on the perception and modeling of listening behavior in a group and the perception of non-addressees, we did not focus on maximizing the number of first party-participants. Therefore, the number of first-party- participants is not sufficient to carry out statistical tests. Nevertheless, some of the answers still provide interesting points of discussions for future work, and we, therefore, report the found tendencies as such. Perceived Affective Understanding was rated comparatively low, which makes sense, as we modeled no affective components. Also, Attentional Allocation was more positively perceived in the robot’s direction in the attentive listener system. Likewise, participants rated our model higher than the baseline model in terms of Perceived Behavioral Interdependence. Interestingly, participants not only believed that they were affected more by the attentive listener system but also that they could affect the attentive listener system more. However, this is, in fact, not true. Participants could affect both systems to the same degree. Co-Presence was the dimension which received the highest ratings overall. Participants had the perception that they were not alone and that the robots perceived their presence. The proposed model was perceived more positively in the robot’s direction, which makes sense as the baseline model should have disappointed participants’ expectations of the visual focus of attention more often. For Perceived Message Understanding ratings of both the proposed model and the baseline model were more pronounced from the participant’s direction. This means that the participants thought they understood the robots better than the robots understood them, particularly the baseline model. The fact that they perceived the proposed model to understand them better indicates that gaze patterns and back-channels by themselves can already make a difference in the perception of message understanding. However, of course, the implementation of further dialogue behavior apart from the ones mentioned above would further improve the ratings.

In addition to the Social Presence questions, participants also indicated, with mean-scores of 3.6 to 2, that they thought the proposed model was the more attentive listener.

#### 5.5.6 Results II: Participant’s Focus of Attention

**FIGURE 8:**
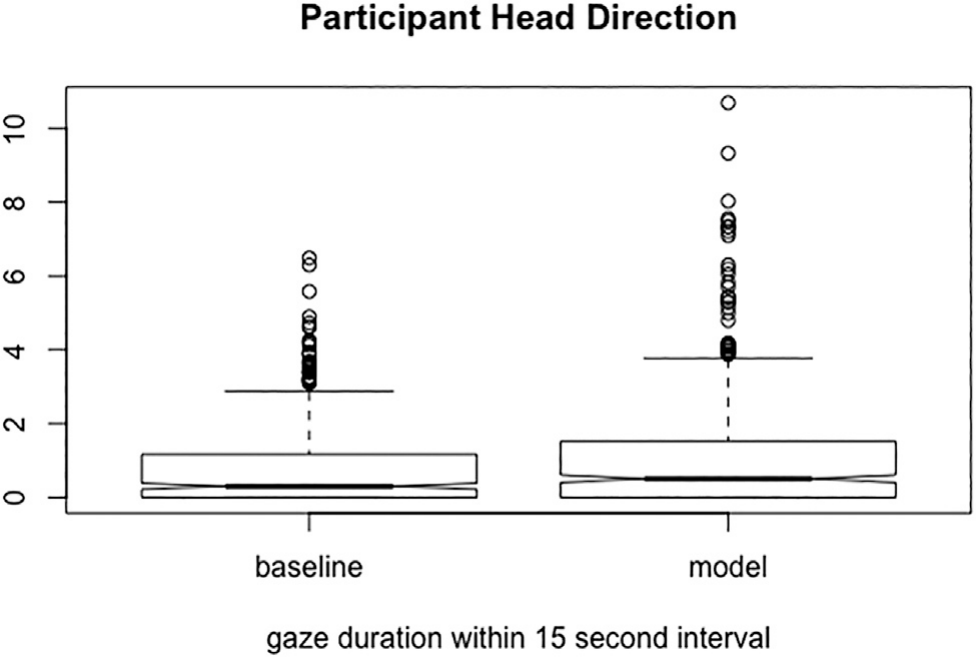
Percentage of time spent gazing towards the attentive listener system robot (model) vs. the baseline system robot (baseline).

Figure 8 shows the time participants spent looking at the robot realizing the behaviors of the attentive listener system vs. the robot realizing the behaviors of the baseline system. It can be observed that the participants spent more time directed towards the robot realizing behavior of the attentive listener system than the baseline system. Finally, a one-way ANOVA revealed that the robot configuration affected the gaze duration of the participant F (1,1028) = 8.554 (*p < 0*.*01*).

### 5.6 Study II: Third Party Observer Ratings

The goal of this study was to investigate third-party observer perceptions of our attentive-listener system. It is, of course, important to evaluate the participant’s impression of our attentive listening system. However, being the speaker, they might also be too preoccupied with talking to notice subtle differences. For this reason, third-party observers might be more suitable to provide a holistic view of the interaction.

#### 5.6.1 Observers

Ratings were distributed over a total of 21 crowd-workers who were recruited via the crowd-sourcing platform crowd-flower. Only English speaking workers from the United States, with the highest trust score level, were included in the study.

#### 5.6.2 Procedure

We set-up a crowd-sourced perception experiment to capture the third-party observer judgements. We adopted elements of the perception studies carried out as in section 4.4. Specifically, we arranged different video shots into one video in such a way that it would reflect the arrangement of the room at the time. As in the human-human experiment, we sampled 30-s video intervals randomly across the interaction with the only constraint that we sampled from both the Cinderalla storytelling sequence as well as the favorite movie retelling part equally. Different from the set-up in the human-human study, we did not ask for the different listener categories (e.g., attentive listener, side-participant, bystander), but asked which participant (of the robots, in this case) they perceived to be more attentive. In total, there were sixty, 30-s videos, five per participant, distributed across the duration of the interaction. There were seven ratings of workers per video segment, which resulted in a total of 420 judgements. As in the human-human experiment a majority voting was implemented to achieve one voting. The video clips were presented to observers in a random manner. We added a time threshold to the task to maximize the quality of ratings. If crowd-workers were clicking too quickly through the sites, they were excluded from the study.

#### 5.6.3 Method

The third and final measure we chose to evaluate our attentive-listener system with, was third-party observer perceptions of attentiveness. Using the perceptions of third-party observers rather than one of the participants has the advantage that third-party observers can concentrate on observing the robot’s behavior without worrying about performing the story-telling task. It was the same methodology we used for both the listener-annotation in the human-human data collection as well as the pre-test.

#### 5.6.4 Results

**FIGURE 9:**
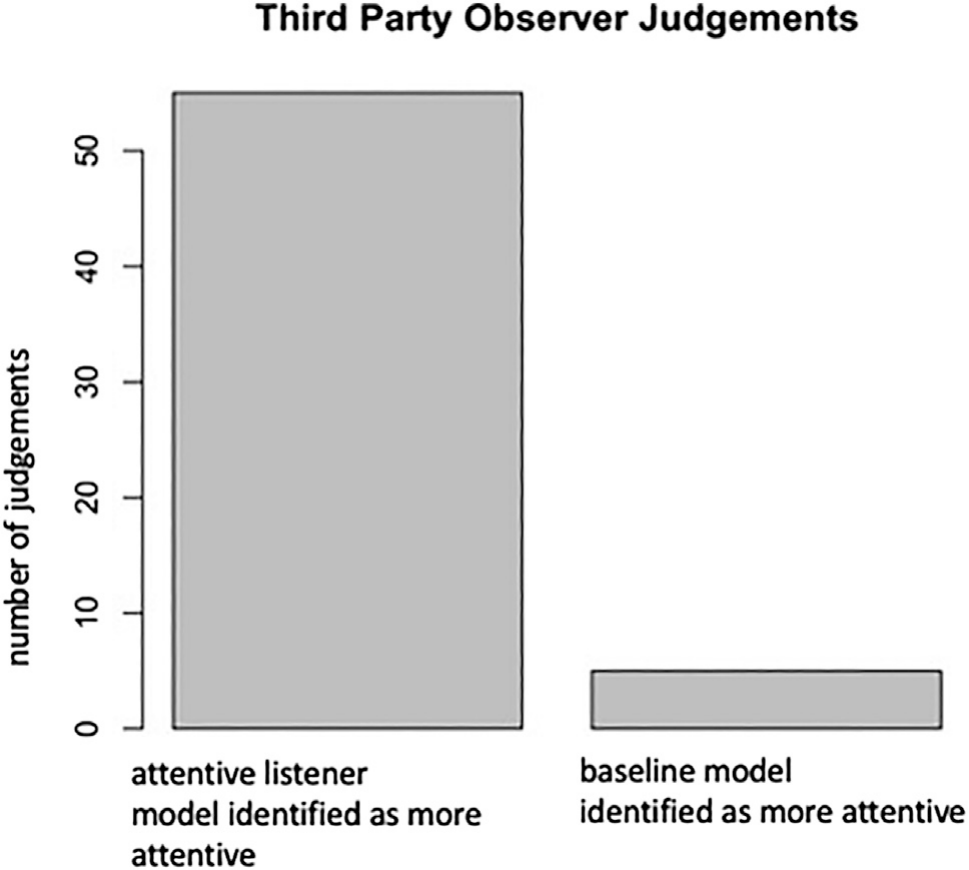
This figure indicates the number of cases in which the third-party observers identified the attentive listener model, respectively, the baseline model as more “attentive.”

As can be seen in Figure 9, observers identified the baseline-model version as the more attentive listener in only five out of 60 cases. A binominal test revealed that the proportion of 0.92 of observers identifying the robot running our attentive listener system as more attentive was significantly higher than the expected 0.5, *p < 0*.*001*.

## 6 Discussion

Listening behavior is essential for the coordination of dialogue in human-human interaction. In human-agent interaction, it has been shown to have a positive effect on rapport (Huang et al., 2011), user engagement (Schroder et al., 2011) and the perceived naturalness of the interaction (Maatman et al., 2005).

Being a listener in a multi-party setting is, however, quite different. The speaker is not as dependent on receiving feedback from one person in particular, as there are several participants he could choose. Similarly also the listeners do not necessarily have to look at the speaker alone.

As can be seen in Figure 3, which shows all gaze comparisons between the different listener categories, both side participant and bystander gaze the most amount of time towards the textitattentive listener. This finding makes intuitive sense as the textitattentive listener would be the listener most engaged in the conversation, and others might want to gauge his reaction towards the speaker.

These findings highlight the importance of considering all participants of a multi-party interaction; not only the speaker and addressee. In fact, in our data collection, gazing at the speaker only account for a maximum of about 50% of the time. This is in line with citealtwang2010don who found that simply increasing the amount of time gazing at the speaker can make gazing behavior appear strange, scary, or awkward. As (Argyle and Cook, 1976; Vertegaal et al., 2001) we also found that the proportionate amount of time the listeners gaze at the speaker is higher than the other way around. We found further proof that the degree of conversational engagement (Oertel and Salvi, 2012; Oertel and Salvi, 2013) is important when modeling eye-gaze. Conversational engagement does not only relate to the speaker but also the different listener categories as gaze is not being distributed equally across the listener categories. Still, participants gaze more towards the listener categories that are related to more engagement. We found only limited support for Mutlu et al. (2009a) finding’s as there were no significant differences between the different listener categories gazing at the speaker, but that it was rather the speaker’s gaze differed in relation to listener category.

In Oertel et al. (2015), we also found that the number of gaze changes did not differ significantly across participant categories. This finding might, at least in part, be related to the scenario chosen for the data collection. The introduction and the PhD pitch were both scenarios which invited the participants to engage in more extended monologues, and it appears logical that a participant would not change its gaze target significantly. We did not carry out a separate analysis looking at gaze changes in the collaborative part of the data collection in comparison the monologue part of the data collection. Therefore, it may well be that differences got canceled out of across conditions.

An additional aspect that might influence the perception of a listener category is the appearance of the speaker. We, therefore, among other reasons, carried out the pre-test, where the appearance of the agent was controlled for. We could show that the differences in the realization of the backchannel did have an impact and also that it made a difference in which modality the backchannel was produced. This finding extended Allwood’s. (2003) finding that different realization of head nods and audio backchannels seem to be also associated with different communicative functions.

It is encouraging to see that our attentive listening system received in general higher ratings in terms of social presence than our baseline system. This is encouraging, especially as greater enjoyment (Richardson and Swan, 2003), performance, satisfaction (Biocca et al., 2001; Tu and McIsaac, 2002) as well as trust (Spencer, 2002), which have been related to the perception of social presence are all essential for developing conversational systems that can engage with people of extended periods of time. Similarly, also the fact that our attentive-listener system was perceived as more “attentive” by third-party observers points towards the same direction. The fact that the robot’s behavior influenced the participant’s focus of attention lends further support to the findings of Mutlu et al. (2009a) and Admoni et al. (2013) who found that the robot’s gazing behavior influences participants behavior and might be used for directing participant roles or turn management. This could particularly important for applications in which the robot is designed to increase, for example, team cohesion.

In conclusion, the main strength of our approach lies in the fact that we not only modeled the gazing behavior towards the speaker but also towards the other listener categories. It is also a first step towards investigating whether a regulating of the degree of attentiveness exhibited towards a specific person has the potential to influence group outcomes as a whole. While explicit intervention is, of course, an effective means, as shown in Matsuyama et al. (2015), more subtle changes of listener behavior might also the potential to achieve similar effects. H2 a,b and c can be accepted.

## 7 Conclusion

This article aimed to build and evaluate an attentive-listener model for multi-party human-robot interaction based on human-human data analysis. We aimed to contribute both to the understanding of human-human listener behavior in multi-party interactions as well as the knowledge on how modeling such behaviors would affect the perception of attentiveness in human-robot interaction.

To achieve this aim, we built upon existing literature on listener categories and used these for data annotations and subsequent analysis. We brought forward a thin-slice approach for collecting annotations by listener category. Results showed that it is feasible to annotate listener categories based on visual information alone.

We used a corpus that used a multitude of sensors such as head-mounted microphones and RGBD sensors. This methodology allowed for the automatic annotation of voice activity, eye gaze and head nods and rendered unnecessary the need for costly and lengthy manual annotations.

Our analysis revealed that there are indeed distinctive gaze patterns that characterize listener categories in multi-party interaction. We found, for example, that the proportion of mutual-gaze and gaze-from-speaker differed significantly across the different categories. We also found differences between listener categories. Our work extends work carried out on dyadic interactions to the multi-party case, making use of substantially more data than used in prior work on listeners. For example, by analyzing the gaze distribution between the speaker and the various listener categories we extended Vertegaal’s (2001) findings.

We implemented an attentive-listening system for multi-party interaction and evaluated it against a baseline system. While the attentive-listening system used the gaze and back-channel distributions stemming from the human-human experiment, the baseline system did not. Instead, it sampled from even distributions with the exception that in case of the speaker looking at a participant for more than 2 s, the participant was assigned the role of attentive-listener. The 2 s threshold was based on practical considerations. Two seconds was too long for it to be a coincidence to be looking at the participant but still short enough to not cause too long of a delay in the system’s reaction. Similarly, also back-channel generation was only varied within the range of possible feedback relevance points provided to both systems. Our analysis showed that humans take on different listener roles within multi-party meetings and vary their feedback behavior accordingly. We found that it is also beneficial for the perception of social presence and attentiveness in a robot, to model listener category-specific feedback behavior.

While our experiments showcase the potential of modeling attentive listener behaviors in multi-party interactions, the current implementation of our attentive-listener system is still limited. While our choice of a story-telling experiment allows us to have more control over the interaction, it is not representative of most real-life scenarios. In most every-day interactions, people would not stay in the role of a listener for such extended periods of time but would be expected to take the turn. In its current implementation, our system does not parse nor manage the content of the dialogue. Neither does it have any dialogue capabilities of its own. Additionally, also, our listener category definitions have its limitations. They are optimized for multi-party situated interaction, rather than for example, a lecturing situation. Similarly, also, the data used for modeling gaze has its limitations. We constrained our data collection by clearing away any unnecessary artifacts that might attract a participant’s focus of attention. We would expect that gaze patterns would differ in situations where participants would be engaged in, for example, a joint assembly task. While the use of different sensor technologies allows us to send the visual focus of attention and speech activity information in real-time to our systems, the set-up is very much restricted to a laboratory setting currently. It would not be transferable to in-the-wild settings. It is of course, already now possible track the head pose of participants in in-the-wild settings and to infer whether a participant’s gaze is directed towards a robot. However, it becomes much more challenging to infer the gaze direction of different participants towards each other, especially in cases where participants are moving around. Additionally, a noisy background would make voice activity detection more challenging. Finally, relying on the extraction of gaze and back-channel related features alone, on the size of data-set available, did not suffice to train a classifier that was able to predict listener categories with high accuracy. Despite these limitations, we believe that we could show the potential of modeling attentive-listener behavior for multi-party human-robot interaction.

## Data Availability

The data used in this article is part of the KTH-IDIAP database, which is available upon request. Requests to access the datasets should be directed to jkgu@kth.se. The specific annotations of the paper are not readily available.
